# “There is no joy in the family anymore”: a mixed-methods study on the experience and impact of maternal mortality on families in Ghana

**DOI:** 10.1186/s12884-022-05006-1

**Published:** 2022-09-05

**Authors:** Emma R. Lawrence, Adu Appiah-Kubi, Hannah R. Lawrence, Maxine Y. Lui, Ruth Owusu-Antwi, Thomas Konney, Cheryl A. Moyer

**Affiliations:** 1 Department of Obstetrics and Gynecology, University of Michigan, Ann Arbor, MI USA; 2 Department of Obstetrics and Gynecology, School of Medicine, University of Health and Allied Sciences, PMB 31, Ho, Volta Region Ghana; 3 School of Psychological Science, Oregon State University, Corvallis, OR USA; 4 College of Engineering, University of Michigan, Ann Arbor, MI USA; 5 School of Medicine and Dentistry, Kwame Nkrumah University of Science and Technology, Kumasi, Ghana; 6 Department of Psychiatry, Komfo Anokye Teaching Hospital, Kumasi, Ghana; 7 Directorate of Obstetrics and Gynecology, Komfo Anokye Teaching Hospital, Kumasi, Ghana

**Keywords:** Maternal death, Maternal mortality, Family, Ghana, LMIC, Mental health, Depression, Complicated grief

## Abstract

**Background:**

Maternal mortality has a multifaceted impact on families, especially in low- and middle-income countries, where rates of maternal mortality are high and resources can be lacking. The objective of this study was to explore the ways that maternal mortality influences the physical and emotional wellbeing, financial stability, and caregiving structure of families, and identifies sources of and gaps in support.

**Methods:**

Our study used a mixed-methods design. All maternal mortalities in an 18-month period at a tertiary hospital in Ghana were identified using death certificates. Participants were 51 family members (either husbands or other heads of households) in families affected by maternal mortality. A questionnaire assessed demographic characteristics and changes in family health, income, and family structure. Two validated scales assessing psychological wellbeing were administered: the Patient Health Questionnaire-9 and the Inventory of Complicated Grief. Semi-structured interviews were conducted to assess impact on family wellbeing.

**Results:**

Quantitative and qualitative results converged to highlight large, negative impacts of maternal mortality on four areas of family wellbeing: 1) mental health and emotional wellbeing; 2) physical health; 3) family structure; 4) financial stability and security. On the Patient Health Questionnaire-9, 54% (27/50) of participants reported elevated depressive symptoms, with 14% (7/50) of scores falling in the moderately severe or severe ranges. On the Inventory of Complicated Grief, 38% (19/50) exceeded the cutoff for significant impairment in functioning. Worsened family health was associated with greater complicated grief (b = 21.41, *p* = .004); there were no other significant predictors of depressive symptom severity or complicated grief. Effects on family health centered on concerns about the nutritional status and health of the surviving infant. Family structure was primarily affected by fracturing of the central family unit by sending children to live with relatives. Immense economic strain resulted from hospital bills, funeral expenses, and loss of income. The majority of participants received helpful support from their family (41/51, 80.4%), the community (32/51, 62.7%), and their religious institution (43/51, 84.3%); however, support often stopped soon after the death.

**Conclusions:**

Maternal mortality has profound negative impacts on families in Ghana. Impacts are experienced by husbands and heads of households, as well as surviving children. Both immediate and sustained support is needed for families following a maternal death, especially mental health and financial support.

**Supplementary Information:**

The online version contains supplementary material available at 10.1186/s12884-022-05006-1.

## Background

Maternal mortality is a devastating consequence of systems failures, gender inequalities, and healthcare disparities [1]. The World Health Organization defines maternal mortality as a death that occurs during pregnancy or within 42 days after delivery or termination of the pregnancy, resulting from any cause related to or worsened by pregnancy [2]. The most common global etiologies of maternal mortality are hemorrhage, hypertensive disorders of pregnancy, sepsis, and complications of abortion [3]. In 1987, the Safe Motherhood Initiative established maternal mortality as a key international health indicator. The subsequent Millennium Development Goals and Sustainable Development Goals have reaffirmed the reduction in maternal mortality as a global priority [4]. Rates of maternal mortality are decreasing globally; however, significant disparities persist. Currently, 94% of all maternal mortalities occur in low- and middle-income Countries (LMICs), with two-thirds occurring in Sub-Saharan Africa [4].

The existing literature on maternal mortality focuses mainly on epidemiological trends, risk factors, and medical management of pregnancy complications. However, maternal mortality also has a devastating impact on husbands, children, and families that often goes unrecognized and unmeasured. Existing studies conducted in select LMICs find that reproductive-age women hold central roles in their households, leading to significant gaps in family support and functioning following their deaths [5–7]. Women are essential to promoting the education and healthcare of their children [8]. Children of mothers who experience a maternal death are significantly more likely to also die in infancy or childhood [5, 9]. Surviving children face increased barriers to accessing quality healthcare [8] and have higher rates of poor health and malnutrition relative to children with living mothers [5, 8]. Increasingly, women also play key roles in financial support and stability of families [7].

Globally, limited research has evaluated the impact of maternal mortality on the mental health and overall wellbeing of families—particularly in Sub-Saharan Africa, where rates of maternal mortality are highest. Identifying potential needs for financial, social, and mental health support could help develop programs and policies that support families affected by maternal mortality. Our study therefore aims to explore the impact of maternal mortality on the physical and emotional wellbeing (including depressive symptoms and complicated grief), financial stability, and caregiving structure of families, and to identify how and from whom individuals receive support and how this support could be improved.

## Methods

This study used a mixed-methods approach to explore the impact of maternal mortality on the physical and emotional wellbeing, financial stability, and caregiving structure of families. Ethical approval was granted by Institutional Review Boards at the Komfo Anokye Teaching Hospital (KATH-IRB/AP/003/20) and the University of Michigan (HUM00175461).

This study was conducted in Ghana, which has a maternal mortality 485 per 100,000 live births [10]. This compares to global average maternal mortality rate of 211 and a high-income country maternal mortality rate of 11 [10]. Leading causes of maternal mortality in Ghana are postpartum hemorrhage, complications of abortion, hypertensive disorders of pregnancy, and infections [11]. Pregnancy care is provided at range of public and private hospitals and clinics, with complicated cases primarily referred to government-run tertiary care hospitals [12]. Ghana’s National Health Insurance Scheme offers universal health insurance coverage during pregnancy, however many medications, supplies, and treatment are not covered by insurance and incur up-front, out-of-pocket expenses [13, 14]. The study site was the Komfo Anokye Teaching Hospital (KATH), which is Ghana’s second largest teaching hospital and serves as a tertiary referral hospital for patients throughout central Ghana. Each year, the maternity unit conducts approximately 10,000 deliveries, of which approximately 100 are complicated by maternal mortalities (unpublished, institutional data).

Participants were husbands in, or heads of households of, families affected by a maternal mortality that was managed at KATH. Additional inclusion criteria were age ≥ 18, fluency in English or Twi, and maternal mortality occurred from June 1, 2019 to December 1, 2020.

All maternal mortalities at KATH in the 18-month period from June 1, 2019 to December 1, 2020 were identified using the WHO definition of maternal mortality [2] and review of death certificate records from the obstetrics and intensive care units. Participants were recruited via telephone 6–12 months following the maternal mortality. Based on the expert opinion of our Ghanaian psychiatrist co-investigator, this time period was selected to balance immediacy of mental health impact with respecting an initial period of grief and transition. To recruit participants, a research assistant called the phone number provided on each death certificate. If the contact person was not the deceased woman's husband, the research assistant requested his name and phone number. If the husband was unable to be contacted or chose not to participate, contact information for the head of household was requested and the survey and interview were carried out with that individual.

Informed written consent was obtained from all participants before any data collection was started. The informed consent and research activities were carried out in either English or Twi (common local language in Kumasi, Ghana) per the participant’s preference. Surveys and interviews were conducted by a trained research assistant in person, at the participant’s home or at another location in the participant’s community, per the participant’s preference. Participants received an incentive of cell phone credit valued at 10 cedis (approximately $2 USD).

First, a study-specific multiple-choice questionnaire was verbally administered to collect demographic information about participants, their families, and the women who died. Additional questions assessed sources and helpfulness of support, changes in family health, income, and family structure. Two validated scales were administered to assess psychological wellbeing. The Patient Health Questionnaire-9 (PHQ-9) is a 9-item scale that assesses presence and degree of depressive symptoms [15]. Individuals rate the degree to which they experienced symptoms of depression (e.g., little interest or pleasure in doing things) over the past two weeks on scales ranging from 0 (Not at all) to 3 (Nearly every day). Potential total scores range from 0 to 27. The PHQ-9 has been used widely in diverse LMIC populations [16, 17], and has been validated in Ghana [18]. Internal consistency of the PHQ-9 was high in the current study (α = 0.86). The Inventory of Complicated Grief (ICG) is a 19-item scale that predicts long-term functional impairment due to grief [19]. Individuals rate how often they experience aspects of complicated grief (e.g., I feel I cannot accept the death of the person who died) on scales ranging from 1 (Never) to 5 (Always). Potential total scores range from 19 to 95. The ICG has previously been used to assess complicated grief in relatives of individuals who died in hospital settings [20]. In the current study, internal consistency of the ICG was strong (α = 0.89).

After surveys were completed, qualitative methods were used to broadly assess the impact of maternal deaths and to allow participants to expand on the themes and experiences that were most important to them. Semi-structured interviews were conducted face-to-face by a research assistant, using an interview guide. The interview guide consisted of open-ended questions about changes in household structure and caregiving structure, economic changes and challenges, and psychological and emotional impact. Open-ended questions were followed by a series of more specific interview prompts to gather more detailed and nuanced information. Interviews lasted between 20 and 60 min, and the entire interview was audio-recorded for subsequent qualitative analysis.

Using a convergent mixed-methods research design, we analyzed quantitative and qualitative data separately and then merged them for comparison [21]. Quantitative survey data was entered into REDCap and downloaded to SPSS (Version 28.0.2.1) for statistical analysis. Basic counts and frequencies were calculated for categorical variables. Responses to psychological scales were summed and categorized per standard scoring guidelines [15, 19]. As exploratory analyses, we evaluated whether factors identified in survey responses and during qualitative interviews were associated with levels of depressive symptoms (measured by PHQ-9) or complicated grief (measured by ICG). Specifically, in separate multivariate linear regression models, we evaluated whether support received (defined as the mean score on items assessing family, community, and religious support); changes in one’s own health compared to before the maternal death; changes in one’s family’s health compared to before the maternal death; changes in the family’s income compared to before the maternal death; and whether there were resources available to support children in the home were associated with 1) PHQ-9 total scores and 2) ICG total scores. Interviews were audio-recorded, translated from Twi to English by a bilingual member of the research team when necessary, and transcribed verbatim. In the event certain words or phrases were difficult to translate, they were left intact in the transcripts. Qualitative analysis was conducted using NVivo 12. First, each transcript was read by two of the researchers, who conducted an independent assessment. Then, the two coders worked together to generate a preliminary list of thematic codes. Next, using an incremental and iterative approach, the two coders reviewed the narrative responses again and developed a codebook of stabilized keyword-phrases. Then, each interview was coded according to the codebook, with weekly discussions regarding any inconsistencies. Finally, using the Attride-Stirling conceptual model, a framework of basic, organizing, and global themes was developed [22].

## Results

Between June 1, 2019 and December 1, 2020, 101 death certificates documenting maternal mortalities were identified. The contact number was called for all 101 maternal mortalities, among which 34 could not be reached. Of the 67 families who were able to be contacted, 16 declined participation. Overall, 51 participants completed the study, of which 51.0% (*n* = 26) were husbands of the deceased woman, 9.8% (*n* = 5) were her parents, 23.5% (*n* = 12) were her siblings, and 15.7% (*n* = 8) were a second-degree relative (cousin, aunt/uncle, grandparent).

**Fig. 1:**
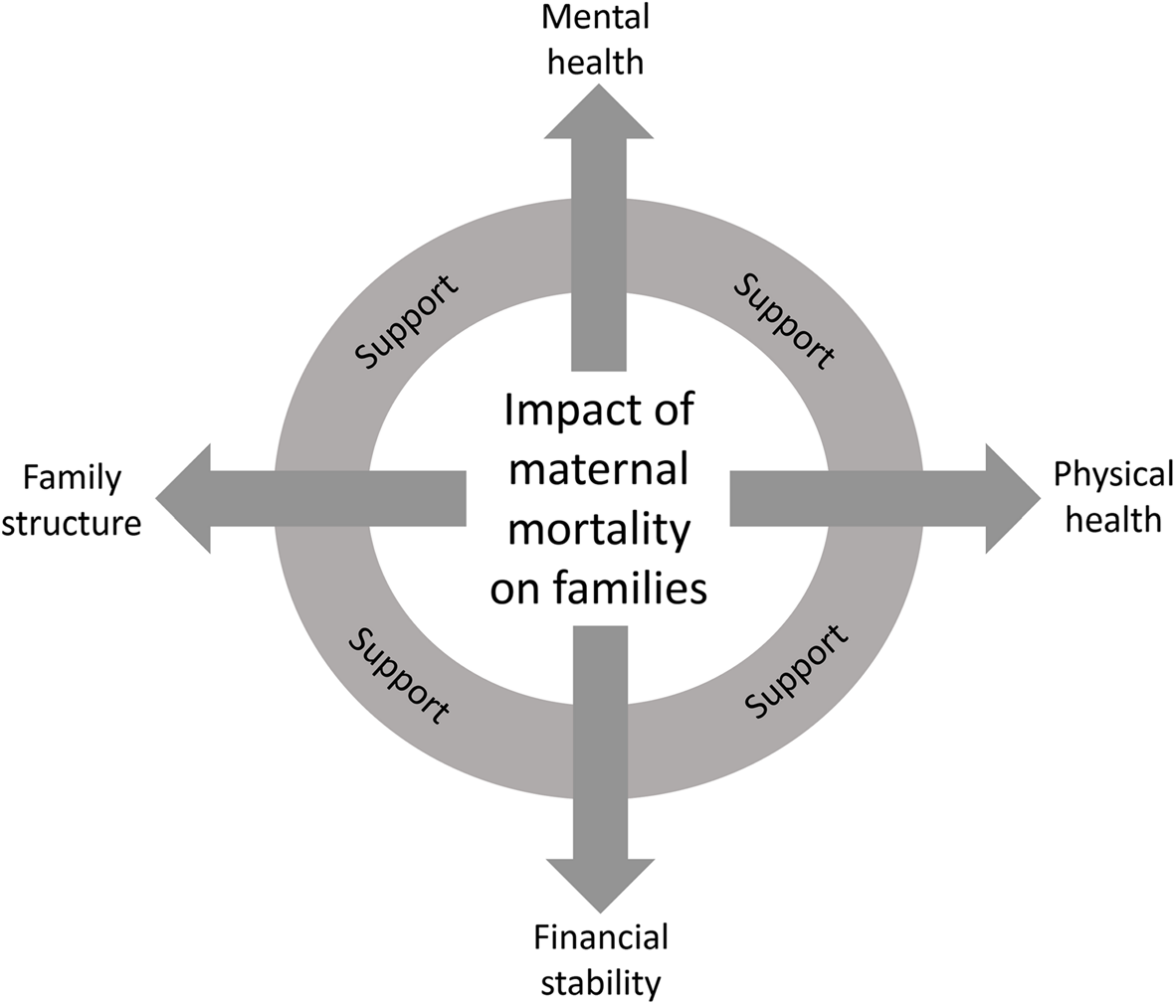
Conceptual model for impacts of maternal mortality on families

Overall, maternal mortality had immense deleterious effects on the surviving family members. Impacts were primarily experienced on four major areas of family wellbeing: 1) mental health and emotional wellbeing; 2) physical health; 3) family structure; and 4) financial stability and security (Fig. 1, Table 1). Support from family members, the community, and religious institutions helped to mitigate these negative impacts; however, support often stopped soon after the death (Table 2). Below, we discuss each area of impact, first outlining quantitative findings then discussing corresponding themes that emerged from qualitative interviews.

**Table 1:** Four main impacts of maternal mortality on families (*n* = 51)

Impact characteristic	*n* (%) or mean (SD)	Representative quotations
**Impact on Mental Health and Emotional Wellbeing**		“There is no happiness in the house… It has brought a huge impact because [as] a young man starting life and this thing has happened to you. It has really affected me emotionally and physically. It has brought a lot of sorrows and sadness. [The children] will be asking ‘daddy, where is mommy, and when is she coming back?’ and the fact is that you cannot tell them the truth.” ID 10, husband, age 43 Ever since she passed there is no joy in the family anymore because the slightest thing my children do reminds me of her ID 9, husband, age 39
PHQ-9 mean score	6.60 (5.97)	
PHQ-9 clinical categories^a^		
Mild depressive symptoms (5–9 points)	14 (28.0)	
Moderate depressive symptoms (10–14 points)	6 (12.0)	
Moderately severe depressive symptoms (15–19 points)	5 (10.0)	
Severe depressive symptoms (20–27 points)	2 (4.0)	
ICG mean score	23.46 (14.58)	
ICG clinical categories		
Significant impairment in functioning (> 25 points)	19 (38)	
**Impact on Physical Health**		“[The woman who died]was the one who was taking the children to the hospital whenever they fall sick.” ID 12, sister, age 40 “She left us a child which we are taking care of… the child [has sickle cell disease] so we are trying to get him treated and we really need help to do that.” ID 6, grandfather, age 69
Participant health now, compared to before the death		
Better	7 (13.7)	
About the same	32 (62.7)	
Worse	12 (23.5)	
Family health now, compared to before the death^b^		
Better	7 (14.3)	
About the same	37 (75.5)	
Worse	5 (10.2)	
**Impact on financial stability**		“It has brought about a huge financial challenge…When she was at the hospital, we were spending every day [on] medicine and blood transfusion…I spent almost what I have saved.” ID 25, husband, age 37
Household income now, compared to before the death^a^		
Lower now	31 (62.0)	
The same	17 (34.0)	
Higher now	2 (4.0)	
**Impact on Family Structure**		“It was too expensive for me and I [couldn’t] support the child for long… so I called my mum to take the child in. At least if the child is with my mum, whatever she eats is what the child will eat. And even if I hadn’t gotten money yet to send for the child’s upkeep I know my mum will take care of [them].” ID 24, husband, age 31 “It has changed a lot of things… Right now I am the only one who is taking care of all the children she left behind. Because of that, I am really going through difficult times. [Deceased wife] was doing all the chores. Now I do it all. I am the one who takes care of [the children]. Everything. I take care of everything.” ID 20, husband, age 48
Status of baby from pregnancy in which the woman died		
Stillbirth	20 (39.2)	
Neonatal/childhood death	7 (13.7)	
Alive	24 (47.1)	
Housing situation of alive babies (*n* = 24)		
Living in same household, full-time	7 (29.2)	
Living in same household, part of the time	3 (12.5)	
Moved to live with another household	14 (58.3)	
Total number of children of the women who died^a^		
Only the baby born during the recent pregnancy	21 (42.0)	
1 other child	9 (18.0)	
2 other children	7 (14.0)	
3 other children	5 (10.0)	
4 other children	3 (6.0)	
5 or more other children	5 (10.0)	
Resources to feed and care for the women’s child(ren) living in the household		
Adequate resources	27 (52.9)	
Inadequate resources	11 (21.6)	
No children living in household	13 (25.5)	

*PHQ-9* Patient Health Questionnaire-9, *ICG* Inventory of Complicated Grief

^a^ Calculated using a denominator of *n* = 50 due to missing data

^b^ Calculated using a denominator of *n* = 49 due to missing data

**Table 2:** Sources and helpfulness of support following a maternal death (*n* = 51)

Source of Support	*n* (%)	Representative Quotations
Family		“Financially, my parents and some family members supported me… And emotionally, that was outstanding from my mom… I think my mom was with me here about nine months. So yeah, the financial support, it was short, but the emotional [was] for a very long time.” ID 29, husband, age 30
Very helpful	18 (35.3)	
Somewhat helpful	23 (45.1)	
Somewhat unhelpful	1 (2.0)	
Very unhelpful	9 (17.6)	
Community		“If you are in a community and the people in the community love you, when you go into difficulty, they will come and support you… After the funeral, the support stopped. “ ID 23, brother, age 40
Very helpful	9 (17.6)	
Somewhat helpful	23 (45.1)	
Somewhat unhelpful	0 (0.0)	
Very unhelpful	19 (37.3)	
Religious		Members of the church came to encourage me and also donated an amount of money to me…I really appreciated what they did for me because in time of need they came to my aid ID 24, husband, age 31
Very helpful	25 (49.0)	
Somewhat helpful	18 (35.3)	
Somewhat unhelpful	0 (0.0)	
Very unhelpful	8 (15.7)	

### Impact on mental health and emotional wellbeing

Overall, participants reported mild levels of depressive symptoms on the PHQ-9, with a total mean score of 6.60 (SD: 5.97). PHQ-9 scores indicated depressive symptoms for 54% of participants (*n* = 27), with 10% (*n* = 5) in the range indicating moderately severe depressive symptoms and 4% (*n* = 2) in the range indicating severe depressive symptoms (Table 1). The model predicting depressive symptom severity was not significant (R^2^ = 0.26, ∆F(5, 30) = 2.06, *p* = 0.10). Main effects of all individual predictors were not significant (all *p* > 0.05; Additional File 1).

On the ICG, participants reported overall moderate levels of complicated grief, with a total mean score of 23.46 (SD: 14.58). Scores for 38% (*n* = 19) of participants exceeded the cutoff of 25 for significant impairment in functioning [14] (Table 1). The model predicting complicated grief was statistically significant (R^2^ = 0.41, ∆F(5, 30) = 4.25, *p* = 0.005). The main effect of changes in family health was significant (b = 21.41, *p* = 0.004) and showed that greater levels of complicated grief was associated with families who had worse health after the maternal death (compared to families with no change or improvement in family health). See Additional File 2 for additional main effects; no other factors were statistically significant predictors of complicated grief.

In qualitative interviews, participants reported overwhelming sadness and emptiness. Many described how being around their children and house constantly remind them of their loss, making it harder for them to move on.

“I grieve a lot because she was a happy person… Anytime I remember her, all I feel is sadness.”

ID 24, husband, age 31

“It has really made us sad because if you remember all that she used to do when she was alive. And also the fact that she has left behind very little children brings about great sorrow.”

ID 3, uncle, age 54

### Impact on physical health

Self-reported current health was excellent for 21.6% of participants (*n* = 11), good for 70.6% of participants (*n* = 36), and poor for 7.8% of participants (*n* = 4). Rates of substance use after the death were low but concerning, with 9.8% (*n* = 5) of participants reporting new or increasing alcohol use and 2.0% (*n* = 1) reporting new or increasing illicit drug use. The majority of participants reported that their own physical health and their family’s overall health was about the same compared to before the death, with 23.5% (*n* = 12) reporting worse personal health and 10.2% (*n* = 5) reporting worse family health (Table 1).

Qualitative data demonstrated that the family’s physical health was impacted in two main ways. First, the woman who died often had previously given birth to other children, and was typically the primary caretaker of these children’s health. This included monitoring their nutrition and growth and taking them to the hospital when sick.

“My wife, she did everything with the kids when she was alive. So she was always the first person the children turned to every time they were in need.”

ID 19, husband, age 30

Second, the surviving newborn was unable to breastfeed, and many participants voiced concern over the comparative cost and quality of formula.

“The baby is not growing well as compared to if the baby was breastfeeding from the mother. But the baby eats in cans [formula], so the growth of the baby is slowing down, and the can food also [is] very expensive and you have to buy it every day.”

ID 25, husband, age 37

### Impact on financial stability and security

For the majority of families, household income decreased after the maternal death with 62.0% (*n* = 31) reporting that household income was lower compared to before the maternal death (Table 1). Qualitative findings reinforced that the death of the mother had a huge negative impact on their household income. Instead of being able to depend on two sources of income, families became dependent on only one—making it harder to sustainably care for the family. Some had to work increased hours to adjust for the loss of a second income.

“The big deal financially is that you [had] someone you are building a future with… She was taking almost the same salary as me, so the two of us, we could have done much more. We could have been financially viable or sustainable over a short period of time. But now, I am alone and there is also a kid to take care of.”

ID 28, husband, age 52

In some cases, the woman’s income had also been essential to support her extended family members. This included supporting elderly parents and paying for school fees for siblings and cousins.

“It has badly affected my finances. Because when she was alive, the little I make from my teaching job she supports me with the money she makes. She was a trader so even when I haven’t received my salary at the end of the month she would support me until my salary comes. She was indeed my helper… The family income has really been impacted, especially from the angle of her siblings and their mother, as well. She was the breadwinner for them… She dedicated most of her earnings and eight years of work to the family. She took one of her siblings to the university then took two to secondary school. So… they’ve lost all of this.”

ID 19, husband, age 30

Several participants even reported losing their job because of too many missed hours from caring for their loved one at the hospital or stopped working to care for their children at home. Others had to work reduced hours that worked around their children's school schedule.

“I was the one taking care of her at the hospital so I was not able to go to work and they dropped me.”

ID 25, husband, age 37

“After her death, I haven’t been able to go look for work for money like I used to because the children are very young. When I wake up in the morning and they have to go to school, I have to do the little things to prepare them for school and take them by the time I get home it would be 9am. I then go to the market but by 2:30 pm I have to stop whatever I am doing and rush home to pick them up from school then take care of them.”

ID 15, husband, age 33

Further, the majority of participants cited that hospital bills and funeral and burial costs have placed a significant strain on their finances. Health insurance did not cover many of the costs, and participants had to rely on their salaries or life savings. Although some received financial help from their family, others held sole responsibility for settling the bills, which led them to take out loans or work extra hours to find the necessary funds.

“It has really affected my finances. When she got sick and was admitted at Komfo Anokye, buying drugs one after the other cost me a lot and the period she spent [in] the hospital also cost me a lot. And even after her death she was not having insurance so it brought to me a huge debt and the funeral was also on me. So at the end of the day, there was a huge reduction in my money.”

ID 18, sister, age 40

Apart from health insurance, no participants reported receiving financial support from the government. Further, participants were universally unaware if financial support programs existed from the government or non-governmental organizations.

### Impact on family structure

Changes in family structure centered around the ability to care for the baby from the recent pregnancy in which the woman died, as well as for her other children. Of the 24 (47.1%) babies who were alive at the time of the study, 58.3% (*n* = 14) were no longer living in the woman’s household (Table 1). Twenty-seven (52.9%) participants had at least one of the woman’s children living in their household, and almost one-quarter (*n* = 11, 21.6%) did not have adequate resources to care for and feed those children (Table 1).

Qualitative findings also emphasized that, for many families, the surviving newborn or other children moved to live with someone else, often a female relative. In addition, most families relocated to a different home. Some reasons for relocating included wanting to restart their lives elsewhere because their current home had too many reminders of their loved one, moving for their new job, or wanting to be closer to their other family members.

“The death of my wife has separated the children and me… After the death of my wife, each of the children are staying with one of my siblings, I just have one child with me… After the burial and everything had settled, I had to move from Kumasi because when anyone sees me or the children, they get sad and it also affected me. Plus everywhere I passed reminded me of her and it was too much to bear so I moved to my hometown where no one knows me to start life again.”

ID 19, husband, age 30

Household duties such as laundry, cleaning, cooking, and caring for the children had commonly been performed by the woman who had died, but after the death, most duties fell on the husband/children's father, with help from other family members such as the children's aunts, uncles, and/or grandparents. In some situations, additional family members moved into the home to help take on some of the household responsibilities that previously had seen done by the women who died. If the children were old enough, they would take on some duties as well.

“The house has now become disorganized because there were certain things she [was] doing but nobody is doing it now… As a man, I was not always in the house, so she was the one doing [household duties] and now their grandmother is the one doing them with the help of my deceased wife younger sister.”

ID 25, husband, age 37

“Now I do everything by myself. The things she would have done if she was here, I now do them myself, be it washing, cooking, whatever, I do myself.”

ID 19, husband, age 30

### Support, and limitations of support

The majority of participants reported receiving helpful support from their family (*n* = 41, 80.4%), the community (*n* = 32, 62.7%), and their religious institution (*n* = 43, 84.3%) (Table 2). However, the degree of overall support received was not significantly associated with levels of depressive symptoms or complicated grief (Additional Files 1 and 2).

Qualitative findings show that family support came from immediate and extended family members and was provided as both emotional and financial support. Many families came together to collectively fund the hospital and funeral costs. Similarly, most interviewed participants reported receiving emotional and/or financial support from their friends, church members, and/or coworkers. Although most support was in the form of those around them checking in, some was in the form of money, clothing, or food donations.

“My family have been very supportive, ever since she got admitted, her drugs and everything they helped me pay for them. They also supported me during the funeral and even after the funeral they had a meeting and decided to help me take care of the children since it would be overwhelming for me and they distributed the children amongst themselves to help me care for them. I talk to the children every day. …with my family the support hasn’t ended. They are still helping….If it hadn’t been for my family I would be dead by now.”

ID 19, husband, age 30

“My pastor helped me. For about two weeks when my wife passed, he was calling me every day. He will call me in the morning, call me in the evening, to advise me. So, he helped me.”

ID 5, husband, age 53

Some participants experienced continued support; however, many expressed disappointment that support waned over time—particularly after the woman’s funeral.

“When she died, we all came together, supported and buried her. But after the funeral, no family member has asked or contributed towards the upkeep of the child she left behind. Everyone is thinking of his or her nuclear family.”

ID 3, uncle, age 54

“The support from the church was very helpful… In the beginning her church was supportive, even bringing items like clothes and others to the children, but they have stopped.”

ID 26, husband, age 29

Among people who supported the participant, the vast majority (*n* = 45, 90.0%) discouraged them from speaking or thinking about the loss. No participants participated in formal therapy or counseling. The primary reason for not participating was a lack of awareness that these resources existed. If offered, some said they would decline these services, while most felt they would have been helpful for sharing experiences and finding comfort in others.

“There was a lack of therapy... and guidance and all that. So, you have to figure that out yourself. So that was one of the challenging things.”

ID 28, husband, age 52

## Discussion

### Main findings

Our findings emphasize that maternal mortality has wide-ranging negative effects on the surviving husbands and heads of households in Ghana. The most significant impacts on family members included mental health and emotional wellbeing, physical health, financial stability and security, and changes in family structure. On the PHQ-9, over half of participants reported at least mild depressive symptoms and on the ICG, over one-third of participants reported complicated grief indicative of significant impairment in functioning. The most notable family physical health impacts included worse nutritional status and health of the surviving infant. Families also experienced large financial losses secondary to the immediate healthcare and funeral costs, as well as longer-term decreases in household income. Changes in family structure reflected the central role that women held in performing household duties and caring for children, with most children moving to live in a different household after the death. Support from family members, the community, and religious institutions helped to mitigate each of these negative impacts. However, qualitative results indicated that support often stopped soon after the maternal death. In the regression analyses, overall support received (defined as the mean score on items assessing family, community, and religious support) was not a significant predictor of levels of depressive symptoms or complicated grief in the surviving husband or head of household.

### Findings in context

Our study highlights that maternal death is associated with mental health difficulties among surviving husbands and heads of households, with qualitative findings indicating emotional distress and quantitative findings showing substantial rates of depressive symptoms and complicated grief. Very limited prior research has been conducted assessing mental health impacts of maternal death in LMICs. A qualitative study done in Kenya reported feelings of grief, frustration, anger, and a sense of loss among family members who experienced a maternal death [23]. A study done in rural China found that three-quarters of husbands experienced posttraumatic stress disorder (PTSD) following the loss of their wife to a maternal death [5]. In our study, decrements in family health were associated with higher complicated grief, but no other indices of personal health, family income, or adequate resources to care for children were significantly associated with depressive symptoms or complicated grief. This suggests that negative impacts on mental health are pervasive across socioeconomic and individual situations and that other, unstudied factors may be associated with higher or lower depressive symptoms or complicated grief following maternal death (e.g., previous mental health history). Support was consistently reported as important and helpful in the qualitative interviews, but participants emphasized that all sources of support were typically short-term and ended soon after the woman’s funeral. Interestingly, helpfulness of support from family, community, and religious sources was also not significantly associated with depressive symptoms or complicated grief. This lack of sustained support may explain why support was not found to be protective when it came to mental health difficulties. Further, formal sources of support were also universally lacking, with no participants aware of or engaged in formal counseling, support groups, or mental health services. This highlights a critical gap in mental health services for husbands and head of households who experience a maternal death, particularly in settings with high rates of maternal death.

Physical health, financial stability, and family structure were all affected by maternal death. First, our study found that physical health concerns were most prominent for the children in the family. Prior research reports significantly higher risks of dying in infancy or childhood among children of mothers who experienced a maternal death compared to those with living mothers [5, 9, 23]. Our study was not designed to assess infant and childhood survival, but our findings suggest etiologies for poor health. Our qualitative findings identified concerns about adequate feeding of the surviving baby, especially regarding the quality and cost of formula as a breastmilk substitute. Further, the woman who died was often the primary caregiver to the children and the primary supporter of their health and development, which included monitoring their growth and seeking healthcare when indicated. These issues are consistent with the literature, which demonstrates nutritional deficits and barriers to accessing healthcare among surviving children [6, 8]. Second, our findings show a large negative impact of maternal death on the financial stability and security of families. In addition to the financial burden of hospital bills, which has been documented in other studies [7], we also found that financial losses are driven by funeral costs, the loss of the woman’s income, and her previous role caring for children, which allowed other family members to work. These findings suggest that financial losses from maternal death may continue over long periods of time following the death. Further, participants received no financial support from the government and had little awareness of programs or resources available. Universally, financial support was desired, especially to help pay for healthcare and education of the remaining children. Third, we found that changes in family structure centered around the ability to care for surviving children, who often moved to live with someone else, usually a female relative. These findings are consistent with studies in Kenya and Tanzania [23, 24] and highlight the central role that women play in the inter-generational functioning of households.

### Strengths and limitations

Strengths of this study include a mixed-methods design, which allows a comprehensive exploration of a nuanced topic. In addition, two reliable and valid psychological scales were used, filling an important gap in the literature by quantifying depressive symptoms and complicated grief in a clinically-actionable manner. Given the depth of qualitative data collected, the research team felt the scope of the study setting was appropriately targeted. Further, since KATH serves as a referral center across central Ghana, participants represented this geographic and sociodemographic diversity. Limitations of this study include it being conducted at a single hospital, which may limit generalizability of findings to other LMICs. While our sample size was quite robust for a qualitative analysis, 51 participants is small number for an inferential quantitative analysis. We recognize that our non-significant findings from the regression analyses may be secondary to the same sample size, and our regression analysis should be viewed as exploratory in nature. A second potential limitation is the scope of participants, which included both husbands and other heads of the household. This may make direct comparisons between participants’ roles more difficult. The authors felt this range of roles reflected the range of impact of maternal deaths on households, and no differences in qualitative themes were noted by role. Further, findings on changes in family health, income, and family structure must be viewed with the understanding that interviews were conducted between six and twelve months following maternal deaths and thus there is variability between participants in the amount of time that has passed. Finally, since rates of depressive symptoms and complicated grief were not assessed prior to maternal death, it remains unknown whether mental health declined across time. Additional research is needed to assess changes in depressive symptoms and complicated grief across the course of maternal health declines and death, and to clarify potential impacts of maternal death on mental wellbeing and psychological distress.

## Conclusions

Overall, we demonstrate immediate and enduring negative impacts on the wellbeing of families who experience a maternal mortality. Primary impacts include high rates of depressive symptoms and complicated grief experienced by the head of household, concerns about nutritional status and health of the surviving infant, immense economic strain on already struggling families, and fracturing of the central family unit by sending children to live with relatives. Participants universally desired additional support, and were unaware if governmental financial and mental healthcare resources existed. The development and awareness of formal mental health and financial support systems should be a priority to mitigate the devastating ways in which maternal death affects the entire family unit.

## Supplementary Information

**Additional file 1.** Linearregression analyses evaluating predictors of depressive symptoms followingmaternal death.

**Additional file 2. **Linear regression analyses evaluating predictors of complicated grief following maternal death.

## Data Availability

The datasets generated and/or analysed during the current study are not publicly available due the fact that full interview transcripts may be personally identifying. The datasets used and/or analyzed during the current study are available from the corresponding author on reasonable request.

## Abbreviations

LMIC: Low- and middle-income country
KATH: Komfo Anokye Teaching Hospital
PHQ-9: Patient Health Questionnaire-9
ICG: Inventory of Complicated Grief
PTSD: Posttraumatic stress disorder
