# Supplementary material for: “There is no joy in the family anymore”: a mixed-methods study on the experience and impact of maternal mortality on families in Ghana
Source: BMC Pregnancy Childbirth. 2022 Sep 5;22:683. doi: 10.1186/s12884-022-05006-1 (PMC9443015; doi:10.1186/s12884-022-05006-1)
Supplement: Supplementary file 1 — Additional file 1. Linearregression analyses evaluating predictors of depressive symptoms followingmaternal death. [file 12884_2022_5006_MOESM1_ESM.docx]

**Additional File 1. Linear regression analyses evaluating predictors of depressive symptoms following maternal death**

| Predictor | *b* (SE) | 95% CI | *t* | *p-value* | *sr^2^* |
| --- | --- | --- | --- | --- | --- |
| Support | -1.50 (1.34) | -4.25, 1.24 | -1.12 | .27 | .03 |
| Own health compared to before | 3.59 (2.37) | -1.25, 8.42 | 1.51 | .14 | .06 |
| Family health compared to before | 2.08 (3.01) | -4.06, 8.22 | 0.69 | .49 | .01 |
| Family income compared to before death | -1.69 (2.34) | -6.47, 3.09 | -0.72 | .48 | .01 |
| Resources to care for children | -4.11 (2.37) | -8.96, 0.73 | -1.74 | .09 | .07 |
