# Supplementary material for: “There is no joy in the family anymore”: a mixed-methods study on the experience and impact of maternal mortality on families in Ghana
Source: BMC Pregnancy Childbirth. 2022 Sep 5;22:683. doi: 10.1186/s12884-022-05006-1 (PMC9443015; doi:10.1186/s12884-022-05006-1)
Supplement: Supplementary file 2 — Additional file 2. Linear regression analyses evaluating predictors of complicated grief following maternal death. [file 12884_2022_5006_MOESM2_ESM.docx]

**Additional File 2. Linear regression analyses evaluating predictors of complicated grief following maternal death**

| Predictor | *b* (SE) | 95% CI | *t* | *p-value* | *sr^2^* |
| --- | --- | --- | --- | --- | --- |
| Support | -6.26 (3.08) | -12.55, 0.04 | -2.03 | .051 | .08 |
| Own health compared to before death | 4.19 (5.43) | -6.90, 15.29 | 0.77 | .45 | .01 |
| Family health compared to before death | 21.41 (6.90) | 7.32, 35.50 | 3.10 | **.004** | .19 |
| Family income compared to before death | -2.81 (5.37) | -13.77, 8.15 | -0.52 | .61 | .01 |
| Resources to care for children | -9.69 (5.44) | -20.80, 1.42 | -1.78 | .09 | .06 |

Bold indicates statistical significance
